# Exploring the genetic basis of anthracnose resistance in Ethiopian sorghum through a genome-wide association study

**DOI:** 10.1186/s12864-024-10545-2

**Published:** 2024-07-08

**Authors:** Chemeda Birhanu, Gezahegn Girma, Firew Mekbib, Habte Nida, Alemu Tirfessa, Dagnachew Lule, Zelalem Bekeko, Getachew Ayana, Tamirat Bejiga, Gudeta Bedada, Meseret Tola, Tokuma Legesse, Habtamu Alemu, Solomon Admasu, Alemnesh Bekele, Tesfaye Mengiste

**Affiliations:** 1 Oromia Agricultural Research Institute, P.O. Box 81265, Addis Ababa, Ethiopia; 2 Department of Botany and Plant Pathology, Purdue University, West Lafayette, IN 47907 USA; 3 Haramaya University, P.O. Box 138, Dire Dawa, Ethiopia; 4 Ethiopian Institute of Agricultural Research, P.O. Box 2003, Addis Ababa, Ethiopia; 5 Agricultural Transformation Institute, P.O. Box 708, Addis Ababa, Ethiopia

**Keywords:** Anthracnose, *Colletotrichum sublineola*, Disease-resistance, Genome-wide association study, *Sorghum bicolor*

## Abstract

**Background:**

Sorghum anthracnose is a major disease that hampers the productivity of the crop globally. The disease is caused by the hemibiotrophic fungal pathogen *Colletotrichum sublineola*. The identification of anthracnose-resistant sorghum genotypes, defining resistance loci and the underlying genes, and their introgression into adapted cultivars are crucial for enhancing productivity. In this study, we conducted field experiments on 358 diverse accessions of Ethiopian sorghum. Quantitative resistance to anthracnose was evaluated at locations characterized by a heavy natural infestation that is suitable for disease resistance screening.

**Results:**

The field-based screening identified 53 accessions that were resistant across locations, while 213 accessions exhibited variable resistance against local pathotypes. Genome-wide association analysis (GWAS) was performed using disease response scores on 329 accessions and 83,861 single nucleotide polymorphisms (SNPs) generated through genotyping-by-sequencing (GBS). We identified 38 loci significantly associated with anthracnose resistance. Interestingly, a subset of these loci harbor genes encoding receptor-like kinases (RLK), nucleotide-binding leucine-rich repeats (NLRs), stress-induced antifungal tyrosine kinase that have been previously implicated in disease resistance. A SNP on chromosome 4 (S04_66140995) and two SNPs on chromosome 2 (S02_75784037, S02_2031925), localized with-in the coding region of genes that encode a putative stress-induced antifungal kinase, an F-Box protein, and Xa21-binding RLK that were strongly associated with anthracnose resistance. We also identified highly significant associations between anthracnose resistance and three SNPs linked to genes (Sobic.002G058400, Sobic.008G156600, Sobic.005G033400) encoding an orthologue of the widely known NLR protein (RPM1), Leucine Rich Repeat family protein, and Heavy Metal Associated domain-containing protein, respectively. Other SNPs linked to predicted immune response genes were also significantly associated with anthracnose resistance.

**Conclusions:**

The sorghum germplasm collections used in the present study are genetically diverse. They harbor potentially useful, yet undiscovered, alleles for anthracnose resistance. This is supported by the identification of novel loci that are enriched for disease resistance regulators such as NLRs, LRKs, Xa21-binding LRK, and antifungal proteins. The genotypic data available for these accessions offer a valuable resource for sorghum breeders to effectively improve the crop. The genomic regions and candidate genes identified can be used to design markers for molecular breeding of sorghum diseases resistance.

**Supplementary Information:**

The online version contains supplementary material available at 10.1186/s12864-024-10545-2.

## Background

Sorghum [*Sorghum bicolor* (L.) Moench] is an important cereal crop adapted to the arid and semi-arid regions of the world. It is a multipurpose crop used for food, feed, and biofuel production. Sorghum anthracnose caused by *Colletotrichum sublineola* is one of the most devastating diseases affecting all above-ground parts of the crop infecting the stalk, foliage, panicle, and grain. The infection results in a decline both in quantity and quality of grain and stover yield [1], leading to losses of up to 50% [2]. Finding a resistance source that is durable could be challenging, given the considerable genetic diversity existing within the pathogen population.

In Ethiopia, sorghum is a widely grown crop that exists in both cultivated and wild forms. The country is considered the center of origin and diversity for the crop [3] contributing germplasms to sorghum breeding programs across the globe. Currently, the Ethiopian Biodiversity Institute (EBI) maintains over 9,000 sorghum accessions [4]. These sorghum germplasm collection exhibits extensive genetic variation which has been explored by sorghum research programs targeting different traits. A few examples of such traits include cold [5] and drought [6–8] tolerance, resistance to anthracnose disease [9], grain mold [10, 11], and agronomic characteristics [12, 13]. Recently, a subset (*n* = 2000) of the accessions from EBI were studied for diverse traits at distinct locations [4]. Comprehensive germplasm characterization and data collection on diverse traits have been undertaken on these accessions. Extensive data were generated and different research findings were published; including a large-scale genome-wide association study on the initial set of 2000 accessions targeting important traits [4]; which was followed by a comprehensive phenotypic and genomic characterization of a core subset (*n* = 387) of the collection [14], and genome-wide association studies for grain mold resistance [10, 11]. The aim of downsizing the collection to a manageable but representative core subset was for ease of detailed characterization of traits [14]. The current study focuses on characterizing the core subset for resistance to anthracnose. During both characterizations of the initial subset and the core subset, the accessions were not studied for resistance to anthracnose. Therefore, the objective of this study was to explore the core subset of accessions for anthracnose resistance through a multi-environment genome-wide association study. Specifically, the study aims at identifying loci that contribute to anthracnose resistance quantitatively.

## Results

### Anthracnose resistance in Ethiopian sorghum germplasm core collection

**Fig. 1:**
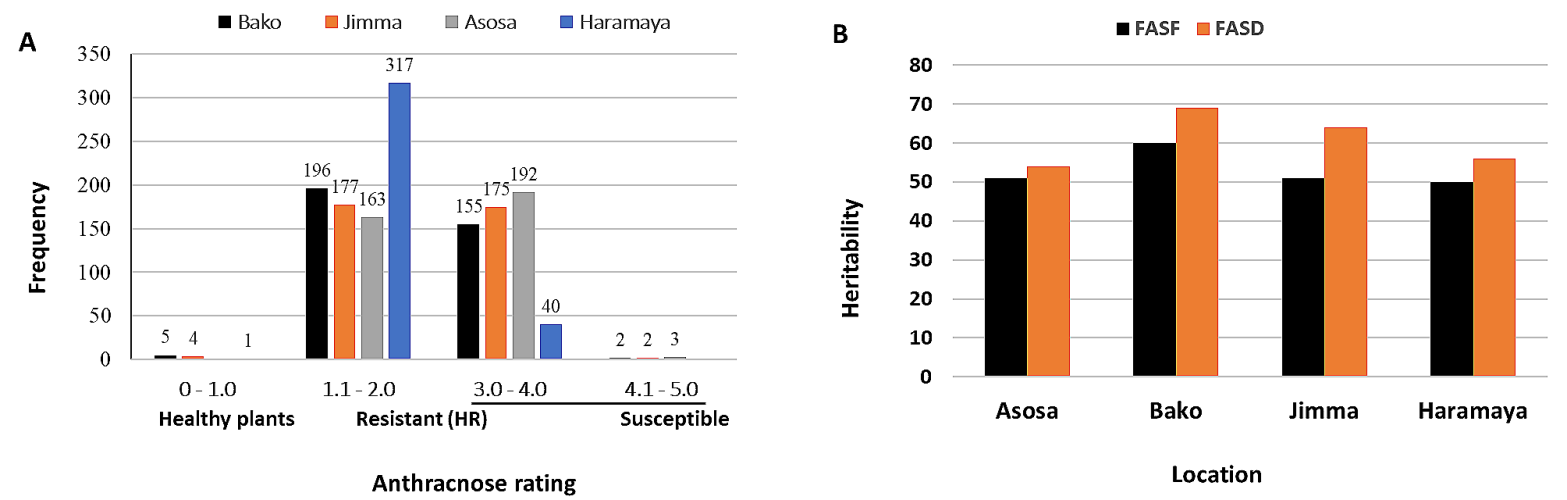
Anthracnose severity rating and frequency distribution across accessions. a Frequency distribution of anthracnose rating across sorghum accessions. b Variation in heritability between testing environments based on anthracnose rating. Anthracnose rating values for each accession are mean from two years where each year’s scores were obtained from plot basis data at the peak of disease severity. A rating scale of 1–5 was used, where 1 and 2 represent resistance, and 3, 4, and 5 are susceptible. The rating scales are described based on the severity as 1 = healthy plant, 2 = hypersensitive reaction with a local lesion, 3 = bottom leaves infected with acervuli, 4 = middle to bottom leaves infected with acervuli, and 5 = whole plant including flag leaf infected with acervuli

Phenotypic analysis revealed that a large number of accessions in this study had hypersensitive reaction (HR) mediated resistance to anthracnose across testing sites (Fig. 1a and Table S1). However, it was observed that responses of genotypes to anthracnose varied across different testing sites. At Bako, Jimma, and Haramaya, a total of only five, four, and one accession, respectively, exhibited resistance without the HR response, each scoring a value of 1. About 196 accessions at Bako and 177 accessions at Jimma had scores between 1.8 and 2.0, indicating that the majority of the accessions exhibited resistance with HR (Table S1). Similarly, at Asosa and Haramaya, 163 and 317 accessions showed HR response to anthracnose, respectively. Among tested accessions, 157, 177, 202, and 40 accessions were susceptible to anthracnose at Bako, Jimma, Asosa, and Haramaya, respectively. The anthracnose resistance response of most genotypes varied depending on locations, with some accessions exhibiting resistance at one location but susceptibility at another. These differences may be associated with variations in pathogen race present at the different testing sites. Among tested genotypes, only 53 genotypes showed consistent resistance response (< 2.0) to anthracnose across locations, whereas 13 genotypes exhibited consistent susceptibility (*≥* 3.0) than the susceptible check (TAM428) (Table S1). We observed that about 30 genotypes were resistant at Bako, Asosa, and Haramaya, but susceptible at Jimma. On the other hand, 50 genotypes showed resistance to anthracnose at Bako, Jimma, and Haramaya, but were susceptible at Asosa. Additionally, 27 genotypes were susceptible at Jimma, while exhibiting a resistance response at Bako, Haramaya, and Asosa. Furthermore, 26 genotypes showed resistance response at Haramaya but found susceptible to anthracnose at Bako, Jimma, and Asosa. We identified three accessions (ETSL 100,332, ETSL 100,476, ETSL 100,761) consistently demonstrating high resistance and two accessions (ETSL 101,640, ETSL 100,544) exhibiting susceptibility at Bako and Jimma (Table S1).

### Heritability

The broad sense heritability (*H*^*2*^) was measured for anthracnose rating for each location. Heritability was estimated based on the individual environment and combined data over years for both field anthracnose severity scores at flowering (FASF) and field anthracnose severity scores at dough stage (FASD) (Table S2 and Fig. 1b). The results of combined data analysis for each location indicated a slight variation in heritability among the testing locations (Fig. 1b). Estimated heritability values at the individual location during each year and combined across years within each location were in general moderate for both FASF and FASD, ranging from 0.51 to 0.69 (Table S2 and Fig. 1b). However, the heritability values for the 2020 cropping season data at Asosa, Bako, and Jimma were relatively low for both scoring methods. At Bako, the heritability estimate for FASD was 0.69 while FASF showed a heritability value of 0.60. At Jimma, heritability for FASD and FASF were 0.64 and 0.51, respectively, while for Asosa and Haramaya, the estimated heritability values for FASD were 0.54 and 0.56, respectively. Heritability for FASF at these two locations was comparable to that of FASD. At all locations, FASD showed higher heritability than FASF. These values are moderate for the anthracnose severity assessment based on quantitative data rating and comparable with those reported previously on sorghum [15].

### Correlation between anthracnose resistance ratings and other traits

Pearson correlation between anthracnose ratings (FASF and FASD), based on whole plot observation, revealed a significant *(P* < 0.001) positive relationship between the two rating methods. The correlation values for these ratings were 0.58, 0.43, 0.49, and 0.51 at Asosa, Bako, Haramaya, and Jimma, respectively. The correlation analysis between FASD and other phenotypic traits, such as panicle weight, panicle yield, and grain yield, displayed a significant (*P* < 0.001) negative relationship between these traits at all locations except at Haramaya (Table 1). These results revealed that anthracnose severity negatively affects the yield and its components. Similarly, a significant and negative relationship was observed between FASD and phenotypic and other growth traits, specifically, days to flowering and plant height which corroborate previous findings [9, 15].

**Table 1:** Pearson correlation coefficients among anthracnose ratings, plant growth, and agronomic traits

	Asosa		Bako		Haramaya		Jimma	
	FASF	FASD	FASF	FASD	FASF	FASD	FASF	FASD
DTF	-0.13*	-0.20***	-0.10ns	-0.36***	0.09ns	-0.04ns	-0.02ns	-0.16**
PHT	-0.12*	-0.17**	-0.07ns	-0.27***	0.10ns	0.01ns	-0.06ns	-0.19***
PWT	-0.14*	-0.16***	-0.11*	-0.20***	0.02ns	-0.01ns	-0.04ns	-0.22***
PY	-0.11*	-0.18***	-0.10ns	-0.15***	-0.02ns	-0.07ns	-0.09ns	-0.29***
GY	-0.10ns	-0.17***	-0.12*	-0.22***	0.04ns	-0.01ns	-0.10ns	-0.29***
FASF		0.58***		0.43***		0.49***		0.51***

*Keys* *, **, *** significant probability level at 0.05, 0.01, 0.001, respectively, ns = non-significant, DTF = days to flowering, PHT = plant height (cm), PWT = panicle weight (g), PY = panicle yield (g), GY = grain yield (Kgha^− 1^), FASF = anthracnose severity scored at flowering, FASD = anthracnose severity scored at dough stages when the disease is at its peak stage

### Principal component analysis

**Fig. 2:**
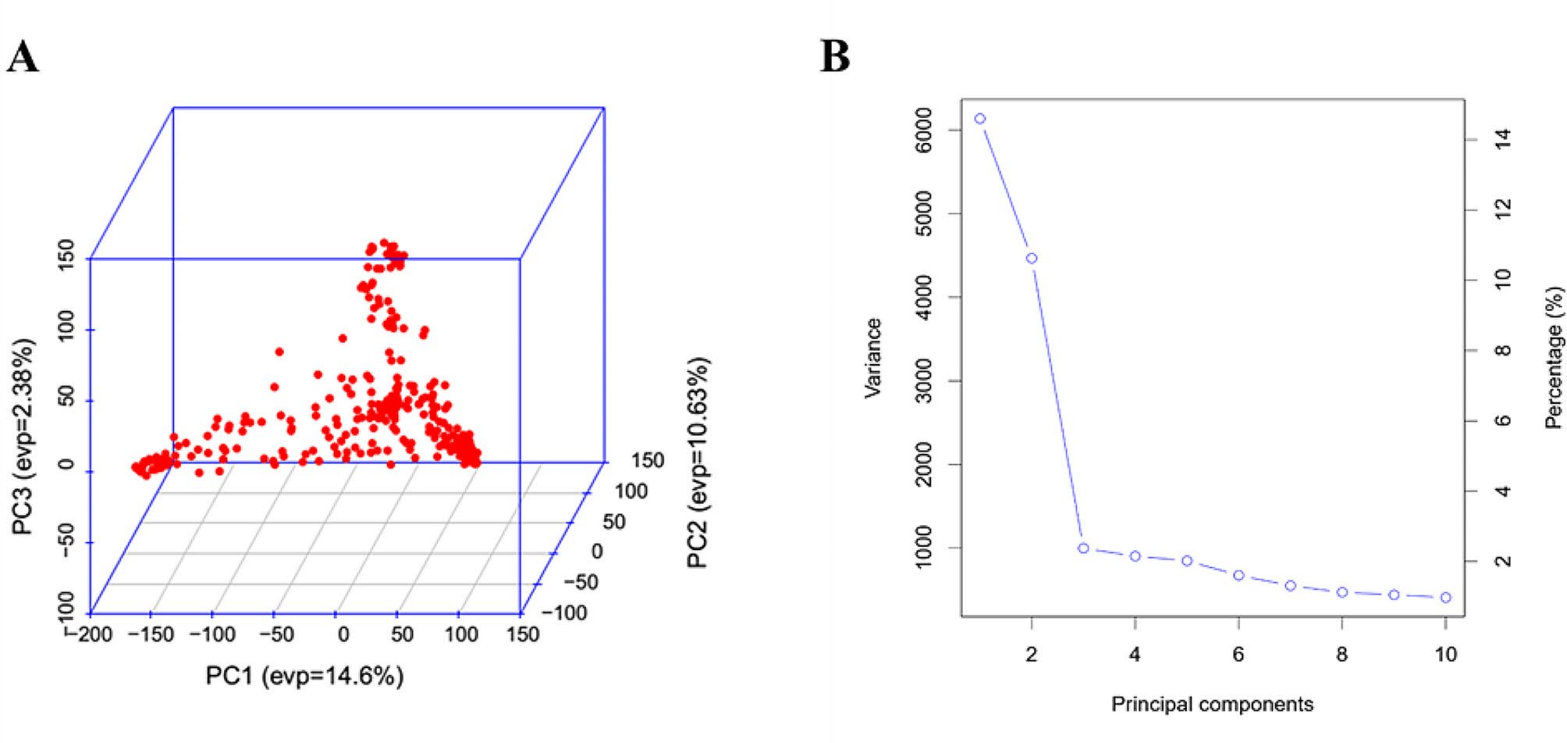
Principal component analyses of 329 Ethiopia sorghum landrace accessions based on 83,861 high-quality SNPs. **a** 3D plot of the first three principal components. **b** A scree plot displaying the first 10 principal components. A large proportion of the variances contained in the data are retained by the first two principal components as indicated on the scree plot

The first three principal components (PCs) elucidated the population stratification within the collection (Fig. 2). A scree plot was generated to visualize how much of the total variance is represented by each of the first 10 principal components. PC1, PC2, and PC3, all together, explained about 27.6% of the overall variance (Fig. 2a and b).

### GWAS for anthracnose resistance

**Fig. 3:**
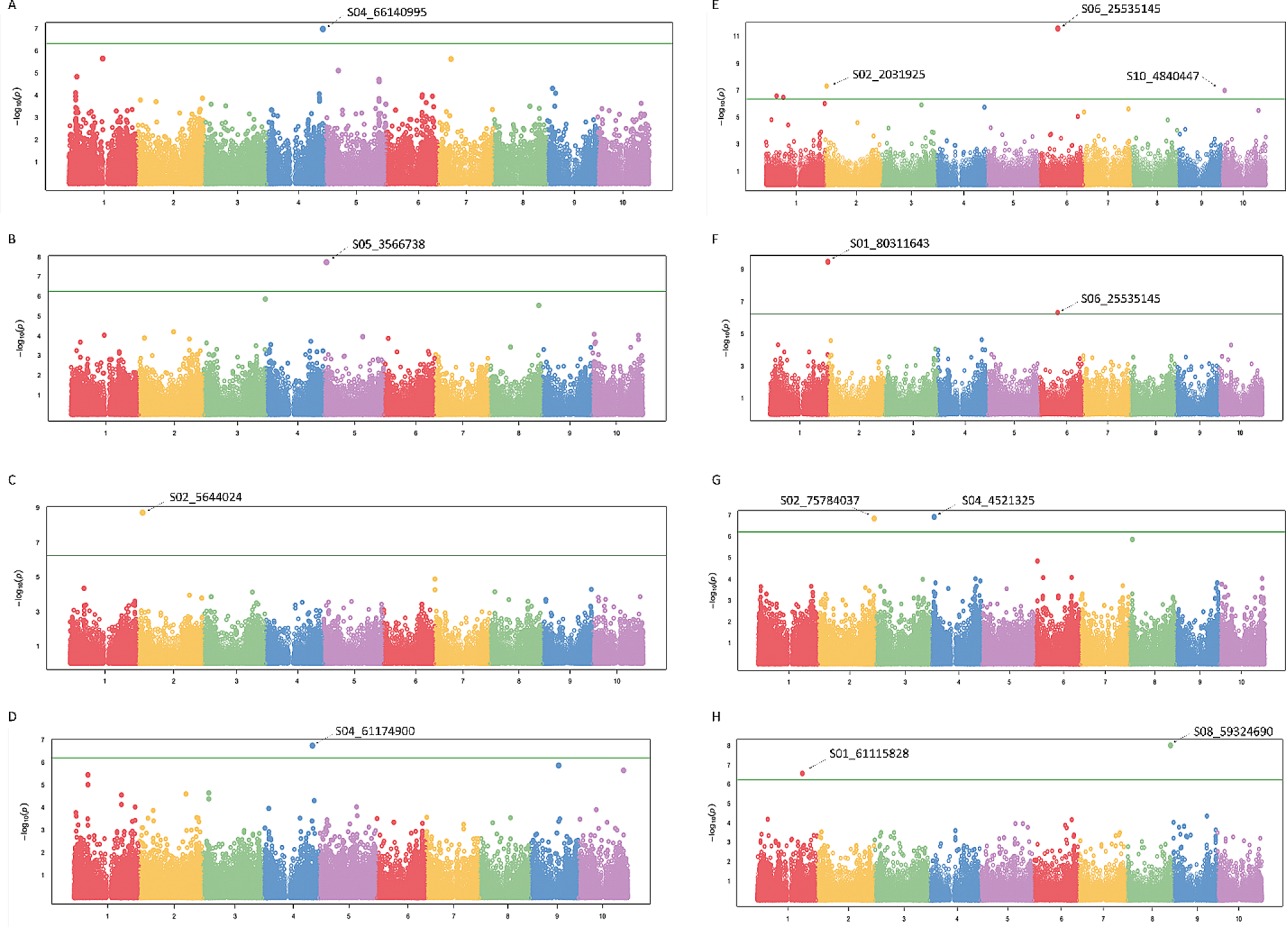
Genome-wide association analysis of anthracnose resistance using different models. SNPs detected from single location-single-year data for FASF at Bako (**A**) and Haramaya (**B**) using BLINK; for FASD at Asosa (**C**) and Haramaya (**D**) using BLINK and MLM, respectively. SNPs detected from combined data at individual locations over years for FASD at Bako (**E**) using FarmCPU; at Bako (**F**) and Jimma (**G**) using BLINK; SNPs detected from combined data across locations for FASF using BLINK (**H**)

The field quantitative disease rating for anthracnose resistance involved assessing disease severity at two different sorghum growth stages: field anthracnose severity scores at flowering (FASF) and field anthracnose severity scores at dough (FASD) stages. These two-stage anthracnose disease rating data were utilized in the GWAS analysis that detected significant SNPs associated with anthracnose resistance (Table 2; Fig. 3, Table S3 and Table S4). The Manhattan plots presented (Fig. 3) are based on the quantitative disease severity score of individual locations in each year, pooled over years in each location and across locations for both FASF and FASD. The GWAS for anthracnose resistance based on individual environment and combined analysis detected a total of 62 significant SNPs for both FASF and FASD. The significant SNPs map to 38 different loci. Of these, 27 and 18 loci were detected for FASD and FASF, respectively while 7 loci were common to both rating methods. Among the 38 loci, individual environment analysis resulted in the highest number of loci detected (24 loci) while combined analysis within each location (combined over years) and combined across all environments (year and location) resulted in 15 and 3 loci, respectively. Moreover, 4 of the loci were commonly detected between individual and combined across environments. Except in a few cases, significant loci were detected for either of the two scoring stages and not for both. For instance, at Jimma in 2021, significant SNPs were detected only for FASD; at Haramaya 2021 for FASF; Haramaya 2022 for FASD, and Asosa 2021 for FASD only. Individual environment analysis for Bako was slightly different, in 2021, significant SNPs were detected for both FASF and FASD. At Bako, 6 loci were detected for FASF and 4 loci for FASD. Of these, two were commonly detected for FASF and FASD. Combined analysis for Jimma also showed similar results. At Jimma, seven loci were detected for FASF and three loci for FASD. Two out of the three significant loci for FASD were also detected for FASF.

**Table 2:** Summary of selected significant SNPs associated with anthracnose resistance

Environment	SNP	Allele	Chr	Position	MAF	Detecting models
Asosa 2021	S02_5644024	C/T	2	5,644,024	0.33	BLINK, CMLM, FarmCPU, GLM & MLM
Bako 2021	S01_80311643	A/G	1	80,311,643	0.42	BLINK & GLM
	S04_66140995	A/C	4	66,140,995	0.07	BLINK, CMLM, GLM & MLM
Bako_combined	S01_23768963	A/G	1	23,768,963	0.16	FarmCPU
	S01_80311643	A/G	1	80,311,643	0.42	BLINK & GLM
	S02_2031925	T/C	2	2,031,925	0.45	FarmCPU
	S06_25535145	A/G	6	25,535,145	0.50	BLINK & FarmCPU
	S10_4840447	T/G	10	4,840,447	0.10	FarmCPU
Haramaya 2021	S05_3566738	T/G	5	3,566,738	0.46	BLINK
	S08_58864910	C/T	8	58,864,910	0.14	CMLM & GLM
Haramaya 2022	S02_57253217	A/G	2	57,253,217	0.21	FarmCPU
	S04_61174900	A/G	4	61,174,900	0.05	BLINK, CMLM, FarmCPU, GLM & MLM
Jimma 2021	S05_2940139	G/A	5	2,940,139	0.36	BLINK
	S10_59803431	A/C	10	59,803,431	0.46	BLINK
Jimma_combined	S02_75784037	G/C	2	75,784,037	0.23	BLINK
	S04_4521325	A/C	4	4,521,325	0.21	BLINK & FarmCPU
	S04_6295203	C/G	4	6,295,203	0.06	FarmCPU
Combined_loc	S01_61115828	C/G	1	61,115,828	0.06	BLINK
	S03_26969328	T/C	3	26,969,328	0.10	FarmCPU
	S08_59324690	A/C	8	59,324,690	0.09	BLINK & FarmCPU

*Key* Bako_combined and Jimma_combined = combined data of 2020 and 2021 cropping seasons; Combined_loc = the combined data across locations

### GWAS using individual location and year data

Based on data from an individual location and year, we could not detect a significant locus associated with anthracnose resistance during the 2020 cropping season. The lower heritability values for the 2020 anthracnose ratings (Table S2) may be the reason for not detecting significant SNPs. However, we identified significant loci associated with anthracnose resistance at Asosa, Bako, and Jimma during 2021 and at Haramaya both during the 2021 and 2022 cropping seasons. Details of significant SNPs are provided in Table 2 and Table S3. We detected a total of 28 SNPs belonging to 24 different loci significantly (FDR < 0.01) associated with anthracnose resistance using data from individual locations in the 2021 and 2022 cropping seasons (Table S3). At Bako, we detected a total of 12 SNPs associated with FASF and FASD. From these, FarmCPU detected three (S01_12967861, S02_77338859, and S05_15711856) for FASF; BLINK detected one (S03_53901112) for FASD whereas GLM detected two (S01_80311643 and S01_80311662) for FASD. The remaining significant loci were detected by more than two models. Using BLINK, CMLM, and GLM models, we detected one SNP on chromosome 1 (S01_39816380) associated with both FASF and FASD. Three closely located SNPs on chromosome 4 were detected for FASF by multiple models. Two of them (S04_66141039 and S04_66141030) were detected by MLM and GLM while the third SNP (S04_66140995, Fig. 3a) was detected by four models (BLINK, CMLM, GLM, and MLM). Another significant SNP closely located to those three is S04_66109867 which was detected for FASF by all the five models. An additional significant locus marked by S07_15368002 is associated with anthracnose resistance and was detected by BLINK and FarmCPU for both FASF and FASD.

At Jimma, seven significant SNPs associated with anthracnose resistance were detected using different models. Three SNPs (S04_65515101, S05_2940139, and S10_59803431) were detected for FASD using BLINK, and two SNPs (S01_68314299 and S05_67756487) for FASD using FarmCPU. S02_75094358 was detected by four models (BLINK, FarmCPU, CMLM, and GLM) whereas S09_58049784 was detected by (BLINK and FarmCP) (Table S3).

At Asosa, a single locus on chromosome 2 (S02_5644024) was detected using five models. The SNP contributed to approximately 12% of the phenotypic variation for anthracnose resistance.

At Haramaya, we detected eight loci significantly associated with anthracnose resistance. Using FarmCPU, we detected four on chromosomes 1, 2, 3 and 10 (S01_58537538, S02_57253217, S03_39160979 and S10_21998625) for FASD. Using BLINK, a SNP on chromosome 5 (S05_3566738) was detected for FASF. Among the SNPs with significant association, S04_61174900 was detected by all five models (BLINK, FarmCPU, CMLM, MLM, and GLM (Table S3). S04_61174900 contributed 2.5 to 4.5% phenotypic variation for anthracnose resistance depending on the GWAS models used. Additionally, S09_35032294 was detected for FASD both by BLINK and FarmCPU, whereas S08_58864910 (PVE = 19.9%) was detected by CMLM and GLM for FASF.

### GWAS using pooled data from each location over years and across locations

By combining data across years at the individual locations and over years and locations, we identified a total of 21 SNPs associated with anthracnose resistance that map to 17 different loci (Table S4). Significant SNPs were detected using the combined year data at the individual locations from Bako, Jimma and Haramaya. Combined analysis of data from Asosa failed to identify significant loci. At Bako, we identified seven significant SNPs associated with FASD on chromosomes 1, 2, 6, and 10 using different models. Based on FarmCPU, we detected five significant SNPs (S01_14446359, S01_23768963, S02_2031925, S06_25535145 and S10_4840447) associated with FASD (Fig. 3e). Using both BLINK and GLM we detected S01_80311643 for FASD. A closely located SNP (S01_80311662) was detected by GLM for FASD that contributed 12% of the phenotypic variation for anthracnose. Both S01_80311643 and S01_80311662 were also detected for FASD by GLM using the 2021 individual location data from Bako. Similarly, S06_25535145 was detected for FASD using BLINK and FarmCPU. The percentage of phenotypic variance explained by the SNP ranged from 9.2% (BLINK) to 10% (FarmCPU).

At Jimma, we identified eight SNPs associated with anthracnose resistance (Table S4). Using FarmCPU, we detected a total of six SNPs on different chromosomes (S02_75094358, S03_65121340, S04_4521325, S04_6295203, S08_59445128, and S09_58049784) for FASF. Using BLINK, we detected the top SNP (S02_75784037) for FASD (Fig. 3g). This SNP explained about 15.8% of the phenotypic variance for the trait. Another significant SNP, S04_4521325, associated with anthracnose resistance, was detected for both FASF (by FarmCPU) and FASD (by BLINK) on chromosome 4. Moreover, a significant SNP(S08_4647621) was detected by GLM (for FASD) and CMLM (for FASF).

At Haramaya, we identified four SNPs significantly associated with anthracnose resistance. Among these, three SNPs (S01_45922176, S01_45922209, and S01_45922219) were detected for FASF by CMLM and GLM models. Meanwhile, a SNP on chromosome 8, S08_59324690, was detected for FASD by four models (BLINK, FarmCPU, CMLM, and GLM).

Using combined data across locations and years (all environments), three significant SNPs were detected for FASF (Table S4). Two SNPs (S01_61115828, S03_26969328) were detected by BLINK and FarmCPU, respectively while the third SNP (S08_59324690) was detected by both models. S08_59324690 also defined a major GWAS peak (Fig. 3h).

### Candidate genes associated with anthracnose resistance

Candidate genes identified within significant loci are presented in Table 3 and Table S5. The total number of genes within local linkage disequilibrium (LD) was determined for the top 12 SNPs (representing 12 loci) based on a 1 Mb region and linkage (r^2^ ≥ 0.1, see methods). Then, the most likely candidate genes linked to or carry the significant SNPs were described based on functional annotation and literature. Consequently, a total of 473 genes were identified which are in LD to the 12 SNPs (Table S5). Of these, 16 genes were identified as having disease resistance function and, therefore, considered the most likely candidates associated with anthracnose resistance (Table 3).

**Table 3:** List of selected significant SNPs, local LD and number of genes in the region associated with anthracnose resistance

SNP	Local LD (kb)	Number of genes in the region	Candidate gene	Distance from SNP (kb)	Description of candidate gene
S01_80311643	281	60	Sobic.001G540300	0	
S02_75784037	415	106	Sobic.002G409200	0	Stress-induced antifungal tyrosine kinase
S02_2031925	23	8	Sobic.002G022300	0	Encodes F-box domain protein
S02_5644024	323	62	Sobic.002G058400	4.9	Sobic.002G058400 encodes a protein homologous to RPM1 (NBS-LRR protein from *Arabidopsis*)
S04_66140995	126	41	Sobic.004G326400	0	Encodes a putative Receptor-Like Kinase Xa21-binding protein 3
S04_61174900	50	17	Sobic.004G267500	1.9	Sobic.004G267500 encodes Putative pathogen-induced protein
S04_4521325	14	3	Sobic.004G056100	0	
S05_2940139	171	36	Sobic.005G033400, Sobic.005G033600, Sobic.005G033700	47, 80, 97	Encodes HMA domain containing protein (HMA domain proteins implicated in binding fungal effector)
S08_4647621	88	24	Sobic.008G047100	0	
S08_58864910	207	40	Sobic.008G156300, Sobic.008G156600, Sobic.008G157400	0, 36, 130	Sobic.008G156300 (Pyridoxal kinase (vitamin B6 kinase)), Sobic.008G156600 (LRR, F-box domain protein), Sobic.008G157400 (NB-ARC disease resistance gene)
S10_4840447	98	29	Sobic.010G061900	0	
S10_59803431	166	47	Sobic.010G261700	0	

*Note* Multiple candidate genes are separated by comma and the corresponding distance from significant SNP and descriptions are also separated by comma accordingly

One of the top SNPs on chromosome 1, S01_80311643, identified based on data from Bako, is located within a predicted gene *Sobic.001G540300*, contributing 8.6–14.1% phenotypic variation for anthracnose resistance but there is no functional annotation for this gene in the database. Another top SNP (S04_66140995) detected by multiple models based on data from the same site is located within the *Sobic.004G326400* gene, accounting for 1.2–50% phenotypic variation for anthracnose resistance. *Sobic.004G326400* encodes a putative Receptor-Like Kinase Xa21-binding protein 3. Moreover, two candidate genes were identified based on a combined analysis of data from Bako. A significant SNP (S02_2031925), detected based on combined analysis at this environment, is located within the coding sequence of the *Sobic.002G022300* gene which encodes an F-box domain protein. Adjacent to *Sobic.002G022300* are *Sobic.002G022000*, *Sobic.002G022100*, and *Sobic.002G022200* which all encode F-box domain protein. Additional candidate genes which are within local LD to the significant SNP include *Sobic.002G021900* and *Sobic.002G022600* which encode wall-associated kinase and AP2-like ethylene-responsive transcription factor, respectively. Another SNP (S10_4840447), detected based on a combined analysis of data from this location is found within the coding region of *Sobic.010G061900* that encodes a pentatricopeptide (PPR) repeat-containing protein.

A SNP, S05_2940139, detected based on data from Jimma is found within *Sobic.005G033000* gene which encodes a protein with similarities to the predicted rice gene *OSIGBa0096F13.7* that encodes UDP-glycosyltransferase. The SNP is located within a local LD region of 171 kb that includes 36 genes. Among these, we identified three candidate genes (*Sobic.005G033400*, *Sobic.005G033600*, and *Sobic.005G033700*) that encode a heavy metal-associated domain-containing protein (HMA). The three genes are located at 47, 80, and 97 kb from the significant SNP, respectively. HMA domain-containing proteins are known to bind fungal effectors [16]. Meanwhile, another SNP (S10_59803431, PVE = 9.1%) detected on chromosome 10 based on data from the same environment is located within the *Sobic.010G261700* gene that encodes a putative LATERAL ROOT PRIMORDIUM 1 (LRP1) protein. Three more candidate genes were identified based on a combined analysis of data from Jimma. A significant SNP, S02_75784037 is found within the *Sobic.002G409200* gene that encodes salt stress response/antifungal protein tyrosine kinase. Another significant SNP (S04_4521325) is located in the coding region of *Sobic.004G056100* which encodes a protein of unknown function. Furthermore, a significant SNP, S08_4647621, was found within the *Sobic.008G047100* gene annotated as an expressed putative protein similar to Pentatricopeptide proteins.

A SNP (S02_5644024) consistently detected at Assosa by multiple models, is located within the *Sobic.002G058300* gene, which encodes an uncharacterized protein. *Sobic.002G058300* is homologous to genes encoding the plant homeodomain (PHD) finger transcription factor which plays a role in chromatin remodeling and transcription regulation [17]. However, the more likely candidate gene at this locus is identified as *Sobic.002G058400*, which is located next to *Sobic.002G058300*. *Sobic.002G058400* is closely located (4.9 kb) to the significant SNP and encodes putative disease resistance protein homologous to RPM1, an NBS-LRR protein from Arabidopsis [18].

Four candidate genes were identified based on data from Haramaya. A SNP (S04_61174900) detected by multiple models in this environment is located at the 5’ UTR region of *Sobic.004G267400*. Adjacent to *Sobic.004G267400*, we identified a more likely predicted candidate gene *Sobic.004G267500*. The two genes share a 3’ UTR region and are transcribed in opposite orientations. *Sobic.004G267500* encodes a protein similar to a putative pathogen-induced protein. *Sobic.004G267500* is located 1.9 kb from the significant SNP. Homologs of Sobic.004G267500 show similarity to sugar utilization and regulatory protein IMP2 from diverse species. Moreover, a SNP (S08_58864910) detected at the same location is found within the intron of *Sobic.008G156300* which encodes a pyridoxal kinase (vitamin B6 kinase). There are 40 genes within local LD to the significant SNP. Of these, there is a more likely candidate gene (*Sobic.008G157400*) located 130 kb from the significant SNP that encodes disease resistance protein. Moreover, a gene (Sobic.008G156600) located at 36 kb from the significant SNP within the LD region is *Sobic.008G156600* which encodes the Leucine Rich Repeat family (LRR) and F-box protein which could be associated with disease resistance.

## Discussion

### Phenotyping, heritability, and correlation analysis

In the current study, we describe results of anthracnose resistance, heritability and correlation obtained from analysis of diverse Ethiopian sorghum germplasm core collections which captures genetic variation from the center of origin and diversity of the crop. Disease assessment across locations was conducted based on quantitative data at two distinct sorghum growth stages (during flowering and post-flowering at the dough stage which is the peak stage of disease severity). The rationale of collecting disease severity data at two different stages was to identify the most prevalent stages of disease and to observe how disease progression varies within different maturity groups. We applied different phenotyping techniques for characterizing traits and various methods for conducting genome-wide association analyses.

The pooled FASD data from each location over the years showed that approximately 45–56% of the tested sorghum accessions exhibited resistance to anthracnose across locations, while 44–55% were susceptible at Bako, Jimma, and Asosa. The data suggest the responses of sorghum accessions to anthracnose at these locations were comparable. These results are consistent with previous reports on Ethiopian sorghum, where a wide range of genetic variation was reported [9, 19]. The identification of genomic regions and specific resistance genes for breeding programs can benefit from this variation. Among the testing sites, Haramaya stands out as the environment where the majority of accessions demonstrated a resistant reaction to anthracnose (Table S1). Recent studies isolated ANTHRACNOSE RESISTANCE GENES (ARG1, ARG2, ARG4, and ARG5) encoding nucleotide-binding leucine-rich repeats (NLR) proteins [20–22]. It is worth noting that the current study identified new loci containing new NLRs and RLKs but none of the ARGs genes were identified showing differences in mapping through biparental and GWAS approaches and differences in field and greenhouse-based genetic screens for disease resistance. As we have described in our previous study [20], there may be accessions that carry resistant alleles at the ARG genes. However, they would be rare (likely less than 1%) to be detected by association analysis. Such rare variants are usually filtered out before the association analysis is conducted because they would have a minor allele frequency (MAF) of less than 5%. The 5% threshold is the standard particularly when low-depth sequencing data are used. Thus, rare variants with a MAF of less than 5% cannot be distinguished from sequencing error. Moreover, the ARGs were identified based on disease assays that use specific pathogen races as opposed to the field based natural infection used in the current report.

The estimate of moderate broad-sense heritability for anthracnose at individual locations indicates that the response of genotypes to anthracnose is influenced by genetic variation but with substantial effects of non-genetic factors. We implemented a two-stage anthracnose disease rating (FASF and FASD) based on field plots under natural infestation (Table S2 and Fig. 1b). The estimation of heritability was improved by utilizing disease ratings at different sorghum growth stages. This suggests that accurate quantification of anthracnose disease at the disease’s peak stage (FASD) is important. A comparable disease rating scenario on sorghum grain mold was reported [11]. The moderate heritability values obtained in the present study from each location indicate that the majority of the observed variation is associated with genetic factors, enabling the detection of candidate loci using GWAS.

We observed significant relationships between anthracnose disease ratings, grain yield, and its components. A significant negative correlation between anthracnose scores and days to flowering indicated that late-maturing plants had reduced anthracnose symptoms. Similar findings were reported previously where both days to flowering and plant height were negatively correlated with anthracnose severity [15]. Our results also highlight a significant (*P* < 0.001) correlation between anthracnose severity ratings for FASF and FASD based on whole plot data which revealed anthracnose severity rating during post-flowering (FASD) to be the most effective predictor of disease severity for making the final decision on resistance to anthracnose. The significant and negative correlations between anthracnose resistance and sorghum grain yield, along with yield-related traits, reveal that the high disease pressure of anthracnose leads to a decrease in yield-related traits and, ultimately, low grain yield. Consistently, a negative and significant correlation between anthracnose severity, grain yield, and head weight was reported [15, 23]. This negative correlation suggests that independent selection would be effective for improving these traits in breeding programs.

### Genome-wide association and identification of candidate genes for anthracnose resistance

Identifying valuable sorghum germplasm speeds up the development of new cultivars and hybrids, sidestepping time-consuming introgression breeding programs for disease resistance, grain, and forage [24]. Conducting an effective genome-wide association study requires a large number of accessions to identify associations between phenotypes and casual loci and alleles. Different genomic studies on Ethiopian sorghum germplasm have revealed valuable genetic diversity, which should be considered as the foundation for sorghum breeding programs in Ethiopia and globally [13, 25, 26]. The large collection of Ethiopian sorghum germplasm (*N* = 1425) previously studied [4], from which the current materials were assembled, possesses valuable genetic diversity for different traits of interest [4, 9–11, 14]. Furthermore, the population structure of the population from which the current accessions were assembled have been described in the previous studies and the materials used here represent a subset of that population [4, 14]. Here, we conducted GWAS for anthracnose resistance using cumulative data from each location and over years, as well as data from a single year at different locations at two sorghum growth stages recorded as FASF and FASD. We employed this multistage disease scoring and conducted GWAS to identify novel loci associated with anthracnose resistance. Five different models (GLM, MLM, CMLM, FarmCPU, and BLINK) were utilized in the GWAS analysis to find SNPs consistently associated with anthracnose resistance. The majority of identified SNPs in our study were commonly detected by BLINK and FarmCPU models. However, only a few of the identified SNPs were deemed significant when employing the GLM, MLM, and CMLM models. This result suggests that employing multi-locus models is efficient for detecting significant SNPs, whereas utilizing an alternative model yielded satisfactory results. Very few significant SNPs were commonly detected for FASF and FASD. Thus, the crop stage at which significant QTLs were detected varied across environments. This can be attributed to variable conditions that favor disease development in different environments (year and location), host responses being modulated by the environment and growth stages as well as differences in pathogen races.

GWAS identified significant SNPs from individual locations in a cropping season, from the combined data in each location over the years as well as the combined data across locations. Our investigation demonstrates that the existing collection of sorghum landraces possesses valuable genetic resources for anthracnose resistance. Similar findings, indicating high genetic variation for anthracnose resistance, were reported on Ethiopian sorghum [9, 19]. The current study also identified different loci associated with anthracnose resistance that offer new avenues for understanding the genetic foundation of anthracnose resistance. In particular, a single SNP located on chromosome 4 (S04_66140995) for FASF was identified by four models, explaining 1.3–50% of the phenotypic variation with different models. This SNP is located within the coding region of *Sobic.004G326400* gene which encodes a putative Receptor-Like Kinase Xa21-binding protein that is required form Xa21-mediated resistance [27]. Another GWAS study on Senegalese sorghum identified a locus carrying protein kinase to be associated with anthracnose resistance [28]. Further, another significant SNP (S02_2031925) on chromosome 2 for FASD was associated with anthracnose resistance. The SNP is located in an intron of Sobic.002G022300 gene that encodes F-box domain protein that have been implicated in the biosynthesis of secondary metabolites in response to plant stresses including diseases and phytohormone signaling. A recent report on anthracnose resistance implicated an F-box protein in mediating an oxidative burst in response to *Colletotrichum sublineola* [29]. Genes encoding an F-box protein, tyrosine kinase, a leucine-rich repeat protein, and peroxidase were identified for anthracnose resistance in the sorghum association panel (SAP) [30]. Other findings on wheat implicated an F-box gene in resistance to wheat leaf rust caused by the fungal pathogen *Puccinia triticina* [31].

GWAS analysis using the FASD identified another locus on chromosome 2 (S02_75784037) to be associated with anthracnose resistance. This locus carries Sobic.002G409200 gene which is annotated to encode a protein tyrosine kinase. In a recent study on sweet sorghum, a protein tyrosine kinase on chromosome 5 was also associated with sorghum anthracnose [24]. In another study on the sorghum association panel [30], tyrosine kinase was identified for association with anthracnose resistance. The top peak SNP detected on chromosome 2 (S02_5644024) by five models based on the data from Asosa is located in the coding region of *Sobic.002G058300* encodes an uncharacterized protein. However, the nearby gene at 4.9 kb, *Sobic.002G058400* encodes a putative disease resistance protein RPM1 which is a likely candidate associated with anthracnose resistance. RPM1 is a nucleotide binding site and leucine-rich repeat protein that functions in pathogen resistance in different plants [32]. Furthermore, another SNP locus, S08_58864910 linked to *Sobic.008G156300* that encodes Pyridoxal kinase (Vitamin B6 kinase) which contributes to disease resistance. In addition, adjacent to this genomic region, at 36 kb from the significant SNP, another gene (*Sobic.008G156600*) which encodes a Leucine Rich Repeat family protein (LRR) and an F-box domain protein was identified. Common bean anthracnose resistance has been associated with LRR receptor-like kinases (RLKs) [33]. Within local LD to S08_58864910 is an NB-ARC disease resistance gene (*Sobic.008G157400*). On chromosome 5, the significant SNP S05_2940139 is linked to three sequence related genes (*Sobic.005G033400, Sobic.005G033600*, and *Sobic.005G033700*) which encode heavy metal associated (HMA) domain protein. Heavy metal-associated domain-containing proteins (HMA) are associated with plant disease resistance [16]. The significant SNPs discussed above have not been previously reported for their association with anthracnose resistance in sorghum. Overall, our GWAS provides valuable insights into the genetic basis of anthracnose resistance in sorghum, provide candidate genes and alleles underlying differences in sorghum responses to against anthracnose.

## Conclusions

Our study assessed sources of resistance to anthracnose through analyses of multi-environment and multi-year comprehensive data of 358 representative accessions of sorghum assembled from Ethiopia. Ethiopia and the surrounding region contain some of the most diverse sorghum germplasm that has contributed valuable traits for improvement. The majority of these diverse accessions display anthracnose resistance which can serve as sources of resistance genes that will be utilized in sorghum resistance breeding. Further, we identified new loci significantly associated with anthracnose resistance, with a subset showing complete resistance with and without the hypersensitive response. The identified loci will serve as valuable genomic resources, offering important genes for resistance breeding, and the foundation for resistance gene discovery and studies on fungal resistance mechanisms. With additional validation, these loci can be effectively utilized for anthracnose resistance breeding using marker-assisted selection, and for establishing crosses by selecting desired parents, gene pyramiding, and trait introgression.

## Materials and methods

### Planting materials

A total of 358 sorghum accessions, comprising 346 landrace collections, four nationally released varieties (Bonsa, Dagim, Adukara, and Asosasa-1), and eight inbred lines from Purdue University were used for this investigation. More than 96% of the landraces are a subset of sorghum accessions from an extensive collection (*N* = 1425) of Ethiopian sorghum germplasms which were previously described [4] and maintained at Ethiopian Biodiversity Institute (EBI) and the national sorghum research program, Ethiopian Institute of Agricultural Research (EIAR). The anthracnose-susceptible genotype, TAM428, was included in the experiment as a standard check. The detailed descriptions of tested materials are presented in Table S6. True-to-type seed sources were used for the study to avoid variability within the accessions.

### The description of the study area

**Fig. 4:**
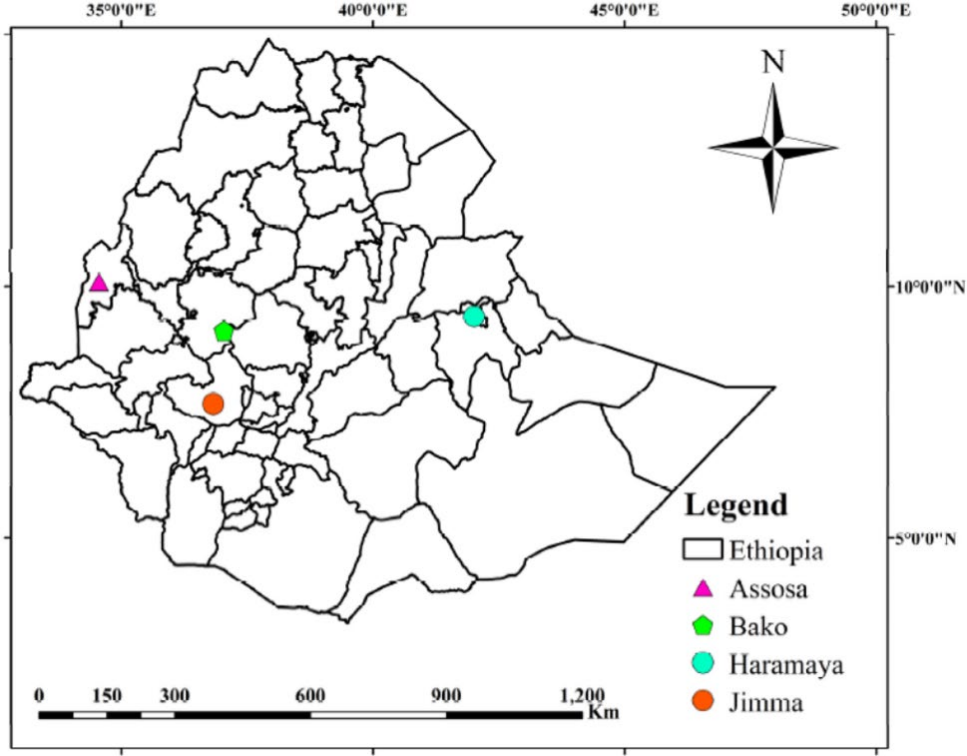
Map of Ethiopia displaying experimental locations

The field experiment for anthracnose resistance screening was conducted in Ethiopia at three locations (Bako, Jimma and Asosa) during the main cropping season of 2020 and at four locations (Bako, Jimma, Asosa and Haramaya) during 2021 and at Harmaya in 2022 (Fig. 4). The specific geographic coordinates for each location are Bako (9° 8′ N latitude and 37°3′ E longitude and 1650 meters above sea level), Jimma (7° 40′ N latitude and 7° 09′ longitude and 1753 meters above sea level), Asosa (10° 03′N latitude and 34° 59′E longitudes and 1580 meters above sea level) and Haramaya (9° 63’ N latitude and 42°05’ E longitude, 2022 m above sea level). These sites represent different agroecological zones (Fig. 4). The brief descriptions of the experimental sites along with the metrological data are presented in Table S7. Among testing locations, Bako, Jimma and Asosa received prolonged rainy months and exhibit warm environmental conditions that favor both foliar and panicle diseases [11] which makes the sites suitable for screening germplasm for disease resistance. The sites are hot spots for foliar diseases that are suitable for screening genotypes under natural infestations. The fourth site, Haramaya, is characterized by a bimodal rainfall pattern with a short rainy season from March to May, while the main rainy season extends from July to September, peaking in August. This region of the country shows a huge potential for sorghum production despite challenges posed by foliar and panicle diseases.

### Experimental design and phenotyping

Two-dimensional spatial positions (rows-columns arrangement) were used to quantify the spatial distribution of anthracnose in the field. All trials were conducted using partially replicated (p-rep) designs as described by [33]. The experimental field was divided into 10 columns (ranges) in which each column consisted of 48 plots (rows). The partially replicated design categorized accessions into two groups that were considered as replications with 30% of the total accessions being replicated. Across all trials, a total of four released varieties (Adukara, Asosa-1, Bonsa, and Dagim), and TAM428 were included as check entries with additional levels of replication. Each accession was planted in a single row of 4 m in length with a 20 cm distance between plants and 75 cm between plots. Sorghum seed was drilled at recommended row spacing (inter-row) and adjusted to 0.2 m spacing between plants (intra-row). Fertilizer was applied at the rate of 100 kg ha^− 1^ NPS at planting and 100 kg ha^− 1^ Urea (half at planting, and half at knee height).

### Disease and agronomic data

The assessment of anthracnose disease was conducted at two growth stages; field anthracnose severity scores at flowering (FASF) and field anthracnose severity scores at dough stage (FASD). The FASD scores represented the stage at the peak of disease pressure. A 1–5 scoring scale was used, where 1 = healthy plant, 2 = hypersensitive reaction with a local lesion, 3 = bottom leaves infected with acervuli, 4 = middle to the bottom leaves infected with acervuli, and 5 = whole plant including flag leaf infected with acervuli as previously described [34, 35]. The rating values of 1 and 2 represented resistance reaction, and 3, 4, and 5 indicate susceptibility. While we attempted to score disease at two sorghum growth stages to observe disease progression, the potential of the accessions for resistance to anthracnose were identified using the final disease score data, i.e., FASD scores at the peak infestation stage of the disease. Phenotypic data on days to flowering (DTF), days to maturity (DTM), plant height (PHT), panicle weight (PWT), panicle yield (PY), and grain yield (GY) were collected as described in Table S8.

### Genotype data

Single nucleotide polymorphisms (SNPs) identified through previous genotyping by sequencing procedure [4] were obtained from the raw data available at the Purdue University Research Repository (10.4231/PYQV-AT79) for the 329 accessions. Among the materials examined (*N* = 358), a subset of 329 accessions with genotype data were used for GWAS. Quality control of the sequence data was performed by removing genotypes with < 20% individual missing rate and minor allele frequency (MAF) values > 0.05 using TASSEL 5.0 [36]. This resulted in a total of 83,861 robust SNPs, which were used for association analysis.

### Data analysis

#### Phenotypic data analysis

The performance of genotypes for anthracnose disease was evaluated based on quantitative field scores at two sorghum growing stages, FASF and FASD. The best linear unbiased prediction (BLUP) values of spatial analysis for anthracnose distribution across the test plots was performed using the SpATS model [37] in R [38].

$$y\; = \;X\beta \; + \;{X_s}{\beta _s}\; + \;{Z_s}s\; + \;{Z_u}u\; + \;{Z_g}g\; + \;e$$

where the vector **y** contains the phenotypic observations arrayed as rows within columns, **β** is a vector of fixed terms including the intercept, a check variety effect, and a resolvable block effect, and **X** is the associated design matrix. The fixed term **Xsβs** and the random component **Z**_**s**_**s** form the mixed model expression of the smooth spatial surface. The vector **u** comprises the mutually independent sub-vectors of random row and column effects accounting for discontinuous field variation. The vector **e** consists of spatially independent residuals.

ANOVA was performed following a linear mixed-effect model using lme4 package [39]. In the analysis, the variance component was estimated for accessions using genotype as random and years as a fixed factor. To classify accessions as either resistant or susceptible, we utilized combined average FASD disease rating data for each location over the years. Accessions with scores *≤* 2.0 were categorized as resistant, while those with scores *≥* 3.0 were considered susceptible as described [34]. We have summarized the anthracnose resistance variability among genotypes using the frequency distributions based on combined anthracnose scoring values of FASD over the years in each location (Fig. 1). Broad sense heritability was calculated as a ratio of variance due to genotypes divided by total variance and it was estimated based on combined data for each location in each year obtained from disease assessments at FASF and FASD. The heritability in single location-single year was calculated:

$${H}^{2}=\frac{{\sigma }_{G}^{2}}{{\sigma }_{P}^{2}}$$

Here, we focused on the estimated heritability obtained from combined data at a single location over the years for FASD at the disease’s peak stage, calculated using the following formula:

$${H}^{2}=\frac{{\sigma }_{g}^{2}}{{\sigma }_{g}^{2}+{\sigma }_{e}^{2}/n}$$

where σ^2^g is the genotypic variance, σ^2^p is phenotypic variance, and σ^2^e is environmental variance, n = number of years.

Pearson correlation analysis between disease severity and agronomic traits was computed using SAS 9.4 version computer software.

#### Principal component analysis

The principal components (PCA) analysis was generated using GAPIT packages during GWAS analysis.

#### Genome-wide association analysis

GWAS was carried out utilizing anthracnose severity scores, FASF and FASD obtained from 329 accessions and 83,861 SNPs. In the analysis, we utilized quantitative diseases rating data for cropping season and the combined data obtained for each location over the years. The best linear unbiased prediction (BLUP) values of spatial analysis using the SpATS model [37] in R [38] were calculated and used for subsequent analysis. We identified a significant association for anthracnose by employing a false discovery rate-adjusted threshold of *p* < 0.05, as executed in the GAPIT. Significant SNP markers were annotated using *sorghum bicolor* 3.1.1 [40] with the physical position in phytozome v13 [41] using J-Browse [42]. The GWAS analysis was conducted using different models including FarmCPU [43], BLINK [44], CMLM [45], and GLM and MLM [46] used in the GAPIT package version 3 [47] in R [38]. We employed these models to find more reliable SNPs loci associated with anthracnose resistance. Local linkage disequilibrium (LD) was determined within a 1 Mb region around significant SNPs. Local LD was calculated using Tassel, where SNPs adjacent to the significant SNP with r^2^ greater than or equal to 0.1 were considered as linked or within local LD. Candidate genes found within this local LD region were selected using the BTx623 reference (v.3.1.1) annotation.

### Electronic supplementary material

Below is the link to the electronic supplementary material.

Supplementary Material 1

Supplementary Material 2

Supplementary Material 3

Supplementary Material 4

Supplementary Material 5

Supplementary Material 6

Supplementary Material 7

Supplementary Material 8

## Data Availability

The manuscript and supplementary files contain all the datasets utilized in this study. GBS raw data is available at the Purdue University Research Repository (10.4231/PYQV-AT79).

## Abbreviations

ANOVA: Analysis of Variance
ARG: Anthracnose Resistance Gene
BLINK: Bayesian-information and Linkage-disequilibrium Iteratively Nested Keyway
BLUP: Best Linear Unbiased Prediction
CMLM: Compressed Mixed Liner Model
FarmCPU: Fixed And Random Model Circulating Probability Unification
FASF: Field Anthracnose severity Score at Flowering
FASD: Field Anthracnose severity Score at Dough
FDR: False Discovery Rate
GAPIT: Genome Association and Prediction Integrated Tool
GBS: Genotyping-By-Sequencing
GLM: General Linear Model
GWAS: Genome-Wide Association Study
HMA: Heavy Metal Associated domain-containing protein
HR: Hypersensitive Reaction
LD: Linkage disequilibrium
LRR: Leucine-Rich Repeat family protein
MATs: Marker Traits Associations
MLM: Mixed Linear Model
NLRs: Nucleotide-binding domain and Leucine-Rich Repeats
QTLs: Quantitative Traits Loci
PCA: Principal Components Analysis
PVE: Phenotypic Variance Explained
RLKs: Receptor-Like Kinases
SAR: Systemic Acquired Resistance
SNP: Single Nucleotide Polymorphism
